# Re-Assessment of the Oral Salt Loading Test Using a New Chemiluminescent Enzyme Immunoassay Based on a Two-Step Sandwich Method to Measure 24-Hour Urine Aldosterone Excretion

**DOI:** 10.3389/fendo.2022.859347

**Published:** 2022-03-21

**Authors:** Yoshinori Ozeki, Mizuki Kinoshita, Shotaro Miyamoto, Yuichi Yoshida, Mitsuhiro Okamoto, Koro Gotoh, Takayuki Masaki, Kengo Kambara, Hirotaka Shibata

**Affiliations:** ^1^ Department of Endocrinology, Metabolism, Rheumatology and Nephrology, Faculty of Medicine, Oita University, Yufu, Japan; ^2^ FUJIFILM Wako Pure Chemical Corporation, Amagasaki, Japan

**Keywords:** primary aldosteronism, 24-hour urine aldosterone level, CLEIA, two-step sandwich method, oral sodium loading test

## Abstract

Since April 2021, the plasma aldosterone concentration has been measured by chemiluminescent enzyme immunoassay (CLEIA) in Japan. In the present study, we developed a new CLEIA using a two-step sandwich method to measure the 24-hour urine aldosterone level. We collected 115 urine samples and measured 24-hour urine aldosterone levels employing radioimmunoassay (RIA), CLEIA, and liquid chromatography–tandem mass spectrometry (LC-MS/MS). The results showed that the 24-hour urine aldosterone levels measured using CLEIA and LC-MS/MS were significantly correlated (ρ = 0.992, P < 0.0001). Based on the results of Passing–Bablok regression analysis, the slope was 0.992 and the intercept –19.3. The 24-hour urine aldosterone levels measured using CLEIA and RIA were also significantly correlated (ρ = 0.905, P < 0.0001). However, the aldosterone level measured by CLEIA was lower than that measured by RIA (slope, 0.729; intercept, 120.9). In Japan, a new guideline for primary aldosteronism has been announced, with changes in the aldosterone measurement method. The cutoff values for oral sodium loading test (OSLT) were changed, but clinical verification using real-world urine samples has not been performed. Therefore, we examined the cut-off value of the 24-hour urine aldosterone level after the OSLT. Receiver operating characteristic analysis revealed a cut-off value for primary aldosteronism of 3 μg/day.

## Introduction

Primary aldosteronism (PA) is widely recognized as the most frequent cause of secondary hypertension, and its prevalence ranges from 5% to 10% in hypertensive patients (1–3). PA is caused by either unilateral hyperaldosteronism (UHA) due to an aldosterone-producing adenoma or bilateral adrenal hyperplasia (4, 5). Patients with PA have an increased risk of cardiovascular events, such as cerebral infarction, myocardial infarction, and chronic kidney disease compared to those with essential hypertension, and early diagnosis makes it a treatable disease (6, 7).

PA is diagnosed by a screening test, followed by a confirmatory or exclusionary test and a subtype test using computed tomography and adrenal vein sampling (AVS). The aldosterone to renin ratio (ARR) is used to screen for PA (8, 9). The number of patients diagnosed with PA has increased due to more frequent screening (10, 11). In Japan, a plasma aldosterone concentration (PAC) > 120 pg/mL, and PAC (pg/mL) to plasma renin activity (ng/mL/h) ratio >200 or a PAC (pg/mL) to active renin concentration (ARC, pg/mL) ratio>40, have been the screening cut-off values for PA (12).

We performed an oral sodium loading test (OSLT), captopril challenge test (CCT), saline infusion test (SIT), and furosemide upright posture test to screen for PA (2–4, 8, 9). When at least one confirmatory test was positive, we diagnosed the patient with PA. When a patient wants to undergo curative adrenal surgery, it is crucial to conduct subtype testing using AVS (2, 3). Measuring of PAC or the urinary aldosterone concentration is important in screening tests, confirmatory tests and AVS. Therefore, an accurate aldosterone measurement method is required for clinical practice.

The PAC and 24-hour urine aldosterone level are conventionally measured by radioimmunoassay (RIA) (13, 14). However, PAC assay kits using the RIA method have been unavailable since March 2021 in Japan. A chemiluminescent enzyme immunoassay (CLEIA) makes it possible to measure aldosterone concentrations more rapidly (12). A relatively strong correlation is observed in aldosterone concentrations between RIA and CLEIA. Some discrepancies in aldosterone concentrations have been reported between the current CLEIA method and liquid chromatography-tandem mass spectrometry (LC-MS/MS), which is the gold standard measurement method according to The Japan Endocrine Society (15). As a result, new CLEIA measurement kits using the sandwich method were developed (16–18). We reported that the PAC measured by the CLEIA using a two-step sandwich method was significantly correlated with the levels measured by LC-MS/MS (17). Therefore, PAC has been measured by the new CLEIA method since April 2021 in Japan.

A screening cut-off value for PA and specific criteria for the confirmatory test may be required to adjust the assay, because the aldosterone levels were lower with the CLEIA method and LC-MS/MS than the RIA method. As a provisional response, the “Japan Endocrine Society Clinical Practice Guideline for the Diagnosis and Management of Primary Aldosteronism 2021” was announced (19). In this guideline, the cut-off values for the PAC and 24-hour urine aldosterone levels using the screening test and confirmatory test changed.

We developed a CLEIA kit using the two-step sandwich method to measure 24-hour urine aldosterone. In this study, we measured 24-hour urine aldosterone levels in patients using RIA, the new CLEIA kit, and LC-MS/MS. The results showed that the 24-hour urine aldosterone levels measured with the new CLEIA kit were highly correlated with those of LC-MS/MS. Similar to PAC, 24-hour urine aldosterone levels measured with the new CLEIA kit were lower than those obtained by RIA. Furthermore, we examined the cut-off value of the 24-hour urine aldosterone level after the OSLT.

## Materials and Methods

### Patients and Study Design

We collected 115 urine samples (60 males and 55 females) from patients hospitalized at Oita University Hospital between October 2018 and July 2020. We diagnosed 48 patients with PA based on a positive screening test and at least one positive confirmatory test for PA (PA group) according to the Japanese Society of Hypertension Guidelines for the Management of Hypertension (JSH 2019) (20). Sixty patients were not diagnosed with PA based on negative screening results for PA (non-PA group). Seven patients did not belong to either group, because they were positive on the screening test, and the confirmatory test had not been performed. The screening test was considered positive when the PAC/ARC ratio was > 40 and the PAC was > 120 pg/mL. The SIT involved intravenous infusion of 2 L of saline over 4 hours, with the patient in a supine position. The test was considered positive when the PAC was > 60 pg/mL after saline loading. The CCT was performed by administering 50 mg captopril, and blood was drawn after 90 min. When the PAC/ARC ratio was > 40 at 90 min after administration, the test was considered positive. The OSLT results were determined by analyzing 24-hour urine samples after the patients had consumed a high salt diet. The test was considered positive when the 24-hour urinary aldosterone and Na excretion levels were > 8 μg/day and > 170 mEq/day, respectively. Urine Na < 170 mEq/day reflected insufficient NaCl loading; those cases were excluded from the analysis. In the present study, 77 of 81 patients with hypertension were taking antihypertensive medications (calcium channel blockers, 65 patients; angiotensin II receptor blockers, 23 patients; a mineralocorticoid receptor antagonist, 4 patients; or others, 14 patients).

All urine samples were collected into a plastic container; the last urine sample was collected after 24 hours. We measured 24-hour urine aldosterone levels in those samples using RIA, CLEIA, and LC-MS/MS.

The study protocol followed the Declaration of Helsinki and was approved by the Ethical Committee of Oita University. All subjects gave informed consent to participate in the study.

### Chemiluminescent Enzyme Immunoassay

We used the Accuraseed Aldosterone S kit (FUJIFILM Wako Pure Chemical Corp., Osaka, Japan) in this study. The analyzer was used “Accuraseed”, an IVD-approved fully automated immunoassay platform in Japan. This analyzer can measure both PAC and ARC in 10 minutes. This assay uses a two-step sandwich method based on the CLEIA, and an antibody that recognizes an immune complex of aldosterone and an anti-aldosterone antibody. We have previously reported that this assay shows a good correlation with LC-MS/MS in serum and plasma samples (17). Detailed verification data for the new reagent appear in the **Supplementary Material**. Urine samples must be pretreated; in this study, they were mixed with 0.2 N hydrochloric acid in a 1:2 ratio and reacted at 25°C for 24 hours to deconjugate glucuronic acid. Subsequently, the hydrochloric acid-treated urine was mixed nine volumes of buffer solution to neutralize it. The urine sample was analyzed in the same way as serum and plasma samples. The aldosterone concentration was determined by multiplying the measured value by 30. The detection range was 3–3,200 pg/mL.

### Radioimmunoassay

We used the SPAC-S Aldosterone kit (Fujirebio Co., Ltd., Tokyo, Japan) in this study. This assay is based on the competitive method. The aldosterone in the sample competes with iodine 125 (tracer)-labeled aldosterone for the antibody coated on the tubes. After aspiration, the level of radioactivity in the tubes is measured with a gamma counter. The degree of binding is inversely proportional to the aldosterone concentration in the sample. Similar to the CLEIA method, urine samples must be pretreated. In particular, the primitive urine sample was mixed with 0.2 N hydrochloric acid at a 1:2 volume ratio and reacted at room temperature for 16–24 hours to deconjugate glucuronic acid. Subsequently, the hydrochloric acid-treated urine was mixed with nine volumes of standard solution to neutralize the urine samples. The urine sample was analyzed in the same way as serum and plasma samples. The aldosterone concentration was calculated by multiplying the measured value by 30.

### LC-MS/MS

As a control, we compared the new assay results with those of LC-MS/MS. We outsourced the LC-MS/MS measurements to ASKA Pharmaceutical Co., Ltd. (Tokyo, Japan).

### Statistical Analysis

The data are presented as mean ± standard deviation and were analyzed using JMP16 software (SAS Institute, Cary, NC, USA). A P-value < 0.05 was considered significant. The correlations were analyzed by Passing–Bablok regression and the Bland–Altman analysis using XLSTAT statistical software (Addinsoft, New York, USA).

## Results

### Baseline Clinical Characteristics of the Patients

The clinical characteristics of the subjects are shown in **Table 1**. Systolic and diastolic blood pressure were significantly higher in the PA than non-PA patients. The PAC and the ARR were significantly higher in PA than non-PA patients, and the ARC was significantly lower.

**Table 1:** Baseline Clinical Characteristics.

	PA (n=48)	Non-PA (n=60)	p
Age (years)	53.8 ± 11.7	54.5 ± 17.1	N.S
Male/Female	22/26	34/26	
BMI (kg/m^2^)	25.5 ± 4.5	27.3 ± 7.1	N.S
Systolic blood pressure (mmHg)	129.8 ± 17.7	119.0 ± 74.3	<0.01
Diastolic blood pressure (mmHg)	80.6 ± 10.3	74.3 ± 12.7	<0.01
HR (bpm)	67.1 ± 10.6	79.4 ± 12.8	N.S
BUN (mg/dL)	13.6 ± 3.8	14.4 ± 4.9	N.S
Cr (mg/dL)	0.8 ± 0.3	0.8 ± 0.3	N.S
K (mmol/L)	3.7 ± 0.5	4.6 ± 0.4	<0.01
eGFR (mL/min/1.73m^2^)	74.5 ± 18.5	70.9 ± 24.0	N.S
Plasma aldosterone concentration (pg/mL)	250.7 ± 141.8	135.3 ± 60.9	<0.01
Active renin concentration (pg/mL)	2.9 ± 1.8	21.3 ± 35.9	<0.01
ARR	115.7 ± 98.4	17.8 ± 16.5	<0.01
Hypertensive medication			
Calcium channel blocker	42/48 (88%)	23/60 (38%)	
Angiotensin II Receptor Blocker	4/48 (8%)	19/60 (32%)	
Mineralocorticoid receptor antagonist	2/48 (4%)	2/60 (3%)	
Others	4/48 (8%)	10/60 (17%)	

Date were shown as Average (mean ± SD).

BMI, Body mass index; PA, Primary aldosteronism.

ARR, The ratio of aldosterone to renin concentrations; N.S, not significant.

Twenty-six patients in the PA group underwent subtype testing using AVS. According to The Japan Endocrine Society, the AVS cut-off values after ACTH stimulation are a lateralized ratio (LR) > 4 and contralateral ratio (CR) < 1 (21). The LR is the ratio of aldosterone to cortisol in the dominant adrenal vein by that in the nondominant adrenal vein. The CR is the ratio of aldosterone to cortisol in the nondominant adrenal vein by that in the inferior vena cava. We diagnosed 16 patients with bilateral hyperaldosteronism (BHA) and 10 with UHA.

### Correlation of 24-Hour Urine Aldosterone Levels Between the CLEIA and LC-MS/MS

This correlation analysis was performed using 115 urine samples. Aldosterone concentrations measured by the CLEIA were significantly correlated with those by LC-MS/MS (ρ = 0.994, P < 0.001).

**Figure 1:**
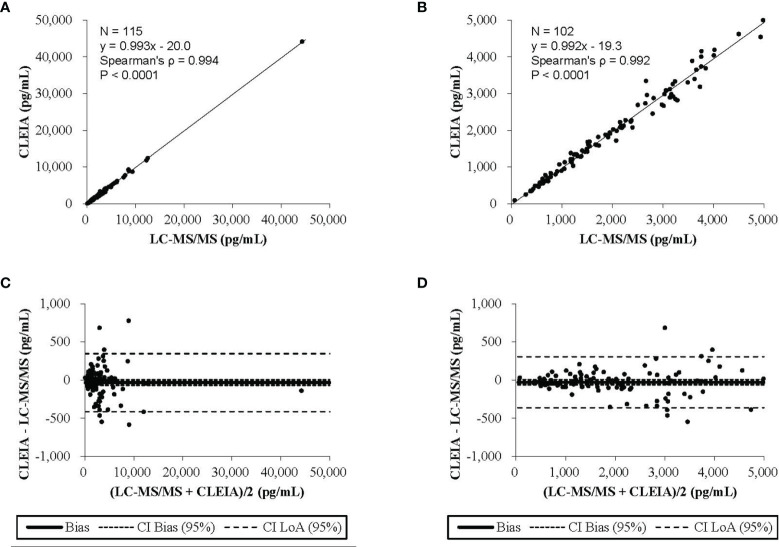
The results of the Passing-Bablok regression analysis and the Bland-Altman analysis **(A)** The CLEIA versus the liquid chromatography-tandem mass spectrometry(LC-MS/MS). **(B)** The CLEIA versus the LC-MS/MS in a concentration range of up to 5000 pg/ml. **(C)** The CLEIA versus the LS-MS/MS. **(D)** The CLEIA versus the LS-MS/MS in a concentration range of up to 5000 pg/ml.

The results of Passing–Bablok regression analysis of the aldosterone concentrations obtained with the CLEIA and LC-MS/MS are shown in **Figure 1A**. The slope was 0.993 and the intercept −20.0, and 95% confidence intervals were also calculated. Bland–Altman analysis showed that the mean difference in aldosterone concentration between the two assays was −33.2 pg/mL, with a 95% confidence interval of −69.0 to 2.6 pg/mL (**Figure 1C**).

To include concentrations of up to 5,000 pg/mL, the analysis was performed using 102 urine samples. Aldosterone concentrations measured by the CLEIA were significantly correlated with those measured by LC-MS/MS (ρ = 0.992, P < 0.0001). The result of Passing–Bablok regression analysis of the aldosterone concentrations obtained with the CLEIA and LC-MS/MS are shown in **Figure 1B**. The slope was 0.992 and the intercept −19.3; 95% confidence intervals were also calculated. Bland–Altman analysis showed that the mean difference in the aldosterone concentration between the two assays was −28.9 pg/mL, with a 95% confidence interval of −62.4 to 4.6 pg/mL (**Figure 1D**).

### Correlation of 24-Hour Urine Aldosterone Levels Between the CLEIA and RIA

This correlation analysis was performed using 88 urine samples. The 24-hour urine aldosterone levels measured by CLEIA were significantly correlated with those determined by RIA (ρ = 0.956, P < 0.0001). The aldosterone levels measured by the CLEIA were lower than those measured by RIA.

**Figure 2:**
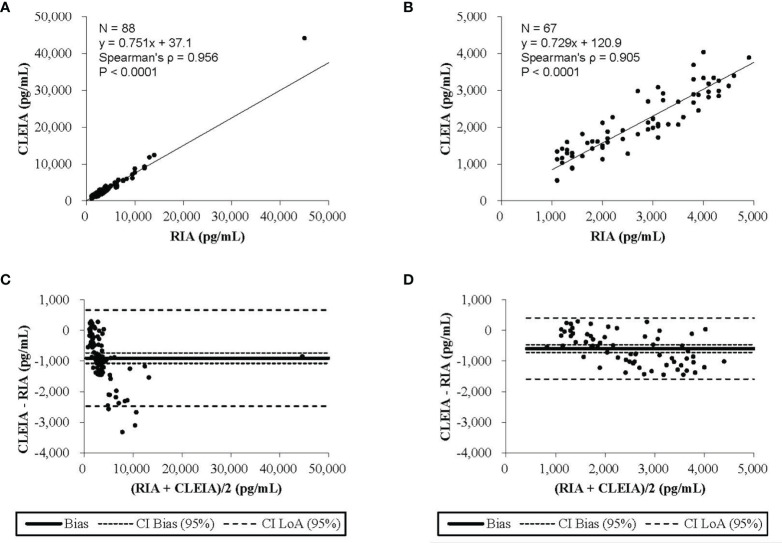
The results of the Passing-Bablok regression analysis and the Blond-Altman analysis. **(A)** The chemiluminescent enzyme immunoassay (CLEIA) versus the radioimmunoassay (RIA). **(B)** The CLEIA versus the RIA in a concentration range of up to 500 pg/ml. **(C)** The CLEIA versus the RIA. **(D)** The CLEIA versus the RIA in a concentration range of up to 5000 pg/ml.

The results of Passing–Bablok regression analysis between the 24-hour urine aldosterone levels determined by the CLEIA and RIA are shown in **Figure 2A**. The slope was 0.751 and the intercept 37.1; 95% confidence intervals were also calculated. Bland–Altman analysis showed that the mean difference in aldosterone concentration between the two assays was −905.2 pg/mL, with a 95% confidence interval of −1,074.7 to −735.6 pg/mL (**Figure 2C**).

At concentrations up to 5,000 pg/mL, the analysis was performed using 67 urine samples. Aldosterone concentrations measured by the CLEIA were significantly correlated with those measured by RIA (ρ = 0.905, P < 0.0001). The results of Passing–Bablok regression analysis of 24-hour urine aldosterone levels determined by the CLEIA and RIA are shown in **Figure 2B**. The slope was 0.729 and the intercept 120.9; 95% confidence intervals were also calculated. Bland–Altman analysis showed that the mean difference in the aldosterone concentration between the two assays was −591.8 pg/mL, with a 95% confidence interval of −716.3 to −467.3 pg/mL (**Figure 2D**).

### Comparison of the 24-Hour Urine Aldosterone Levels After the OSLT Between PA and Non-PA Groups Using the CLEIA

**Figure 3:**
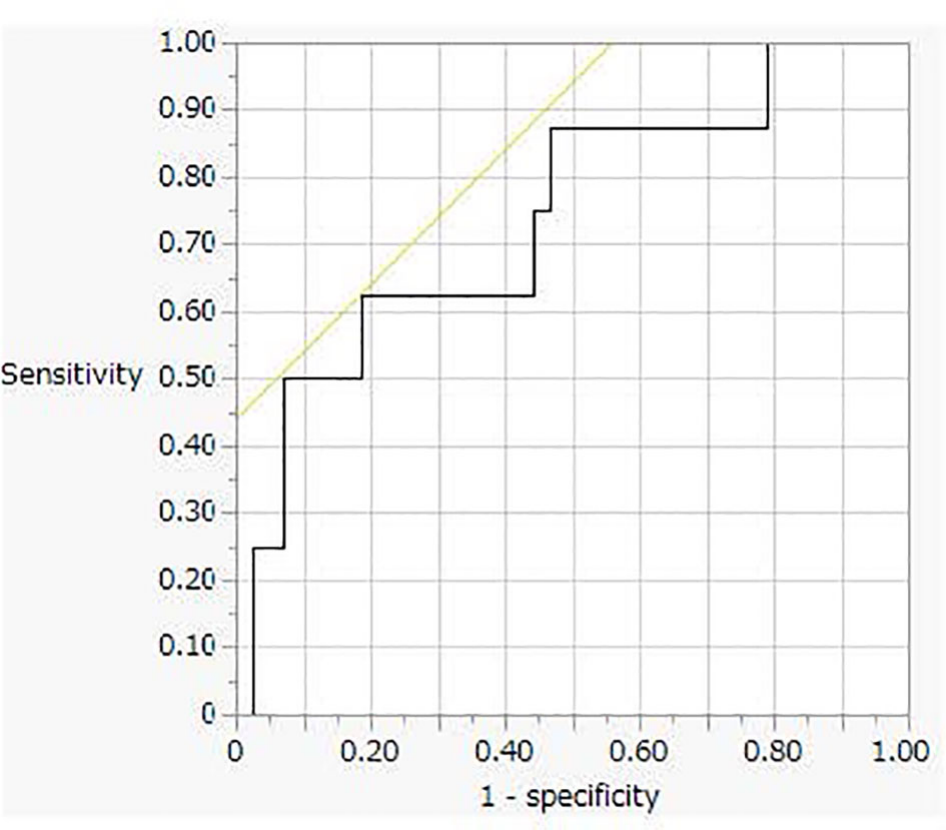
The receiver operating characteristic (ROC) curve analysis for oral sodium loading test cut off value.

This analysis was performed using 51 urine samples (8 PA and 43 non-PA samples, respectively) from patients who had Na excretion levels > 170 mEq/day. Receiver operating characteristic (ROC) curve analysis revealed a cut-off value for the PA diagnosis of 3.1 μg/day (sensitivity, 63%; specificity, 82%, **Figure 3**).

## Discussion

We recently developed a new CLEIA using a two-step sandwich method to measure plasma and serum aldosterone concentrations. PAC has been measured by CLEIA using the sandwich method since April 2021 in Japan.

As was true of based on the changes of the measuring method, the cutoff value for the SIT has been changed to 12 pg/mL from 60 pg/mL (17, 19). But, the recent study showed that the cutoff value for the SIT in the seated position was PAC > 61.6 pg/mL, which was measured by high-performance liquid chromatography mass spectrometry (22). The reason why our assay indicates much lower cut-off value than the previous report might be due to the different assay methods. Our new CLEIA uses double sandwich antibodies, whereas the former report uses single antibody for CLEIA.

In the present study, we verified the utility of a new CLEIA method that measures urine aldosterone excretion. Several studies have shown that the PAC and 24 hour urine aldosterone concentration determined by the CLEIA using the sandwich method was highly correlated with the LC-MS/MS value but lower than the RIA value (16, 17). This study demonstrated that 24-hour urine aldosterone levels measured by CLEIA using a two-step sandwich method can be used in clinical practice, but the cut-off 24 hour urine aldosterone level of the OSLT must be adjusted for the new method.

We collected 115 urine samples (PA group, 48 patients; non-PA group, 60 patients; other group, 7 patients). We confirmed that both the PAC and ARR levels were significantly higher in PA than non-PA patients, and that the ARC was significantly lower.

PA management should proceed step-wise from screening to confirmatory testing, subtype testing, and finally treatment. The OSLT, a confirmatory test for PA, can be performed by measuring the 24-hour urine aldosterone level and NaCl excretion. According to the Japanese Society of Hypertension Clinical Practice Guideline 2019, PA is present when the 24-hour urine aldosterone and Na excretion levels exceed 8 μg and 170 mEq, respectively. In the new Japanese guidelines for PA, the cut-off level of 24-hour urine aldosterone excretion was revised to > 6 μg/day from > 8 μg/day based on the changes in the aldosterone measurement method. However, this cut-off value is tentative; it has not been validated using clinical samples. We therefore investigated 24-hour urine samples from both PA and non-PA patients. In this study, only 20 of the 43 urine samples collected after the OSLT tested positive (PAC ≥ 6 μg/day) by the CLEIA method. The ROC analysis revealed a cut-off value of 3 μg/day with 63% sensitivity and 82% specificity, which was lower than that of the new guidelines.

Second, OSLT is recommended as a confirmatory test for PA according to the clinical practice guidelines of both the Endocrine Society and the Japanese Society of Hypertension (20, 23). The OSLT has both advantages and disadvantages. As the OSLT measures 24-hour urine aldosterone excretion, it reflects that severity of PA. Moreover, we found that a positive OSLT result was significantly associated with both higher 24-hour blood pressure and albuminuria (24). We therefore consider that the OSLT not only serves as a PA confirmatory test but also reflects the organ damage caused by PA. However, the OSLT is cumbersome, and 24-hour urine collection is required. Also, when the 24-hour urine Na excretion level is < 170 mEq, the OSLT result is cannot be evaluated because of a lack of salt loading. It is crucial to educate patients to consume more salt than usual prior to undergoing the OSLT.

Third, UHA, an important pathology of PA, is attributable principally to aldosterone-producing adenomas. Patients with unilateral aldosterone-producing adenomas often present with resistant hypertension, a markedly high PAC, and spontaneous hypokalemia; patients with BHA usually present with mild hypertension, a normal serum potassium level, and a normal-to-high PAC. As UHA is curable *via* unilateral adrenalectomy, it is essential to effectively distinguish UHA from BHA. After diagnosing PA with a confirmatory test, AVS is the gold standard for diagnosing the PA subtype (25–27). We diagnosed 16 patients with BHA, and 10 with UHA employing AVS. The clinical characteristics of the BHA and UHA patients are listed in **Table 2**.

**Table 2:** Baseline Clinical Characteristics of the patients with bilateral hyperaldosteronism and unilateral hyperaldosteronism.

	BHA (n=16)	UHA (n=10)	p
Age (years)	49.6 ± 9.7	53.3 ± 13.3	N.S
Male/Female	9/7	5/5	
BMI (kg/m^2^)	25.6 ± 4.3	24.0 ± 3.4	N.S
Systolic blood pressure (mmHg)	130.1 ± 14.5	133.3 ± 18.5	N.S
Diastolic blood pressure (mmHg)	82.8 ± 7.5	79.7 ± 11.3	N.S
HR (bpm)	69.5 ± 10.4	66.5 ± 8.9	N.S
BUN (mg/dL)	13.7 ± 2.8	12.6 ± 3.6	N.S
Cr (mg/dL)	0.8 ± 0.2	0.7 ± 0.3	N.S
K (mmol/L)	3.9 ± 0.5	3.3 ± 0.5	<0.01
eGFR (mL/min/1.73m^2^)	73.3 ± 14.0	82.2 ± 21.8	N.S
Plasma aldosterone concentration (pg/mL)	229.0 ± 73.5	401.8 ± 184.5	<0.01
Active renin concentration (pg/mL)	2.7 ± 2.0	2.1 ± 1.9	N.S
ARR	120.6 ± 71.3	410.9 ± 420.7	N.S
24-hour urine aldosterone levels (μg/day)	7.2 ± 4.2	13.8 ± 10.9	N.S

Date were shown as Average (mean ± SD).

BMI, Body mass index; ARR, The ratio of aldosterone to renin concentrations.

BHA, bilateral hyperaldosteronism; UHA, unilateral hyperaldosteronism.

N.S, not significant.

Several studies have shown that both confirmatory testing as well as AVS can be avoided in full-blown UHA patients with a markedly high PAC, a reduced plasma renin level, spontaneous hypokalemia, and a unilateral hypodense adrenal tumor (28–30). The 24-hour urinary aldosterone level reflected the total daily level of aldosterone secretion and is useful when evaluating PA pathology (31). In this study, ROC analysis revealed a cut-off value after the OSLT for both UHA and BHA of 9.4 μg/day, with 70% sensitivity and 81% specificity. The calculated positive and negative predictive values for diagnosing UHA were 64% and 80%, respectively. Therefore, the 24-hour urine aldosterone assay may be one of the method of UHA diagnosis.

In conclusion, we re-assessed the OSLT cut-off values in patients with PA using a new CLEIA method to measure urine aldosterone excretion. The cut-off level of 24-hour urine aldosterone excretion for a positive OSLT is 3 μg when the 24-hour urine Na level exceeds 170 mEq.

The new CLEIA aldosterone assay can handle serum, plasma and urine samples, and it is automated, reproducible, and rapid in terms of PA diagnosis.

### Limitations

The present study had several limitations. First, the number of cases was small, particularly the number of patients in the PA group with Na excretion levels > 170 mEq/day. Second, many patients in the non-PA group were on drugs that affected aldosterone levels. These limitations should be addressed in a future study.

## Data Availability Statement

The original contributions presented in the study are included in the article/**Supplementary Material**. Further inquiries can be directed to the corresponding author.

## Ethics Statement

The studies involving human participants were reviewed and approved by the Ethical Committee of Oita University. The patients/participants provided their written informed consent to participate in this study. Written informed consent was obtained from the individual(s) for the publication of any potentially identifiable images or data included in this article.

## Author Contributions

Methodology, YO, TM, and HS. Formal Analysis, YO and TM. Investigation, YO, MK, SM, and YY. Data curation YO, MK, and TM. Writing—original draft preparation, YO, MO, and KG. Visualization, YO and KK. Review and Editing, YO and HS. All authors contributed to the article and approved the submitted version.

## Conflict of Interest

Author KK is employed by FUJIFILM Wako Pure Chemical Corporation.

The remaining authors declare that the research was conducted in the absence of any commercial or financial relationships that could be construed as a potential conflict of interest.

## Publisher’s Note

All claims expressed in this article are solely those of the authors and do not necessarily represent those of their affiliated organizations, or those of the publisher, the editors and the reviewers. Any product that may be evaluated in this article, or claim that may be made by its manufacturer, is not guaranteed or endorsed by the publisher.
