# Supplementary material for: Re-Assessment of the Oral Salt Loading Test Using a New Chemiluminescent Enzyme Immunoassay Based on a Two-Step Sandwich Method to Measure 24-Hour Urine Aldosterone Excretion
Source: Front Endocrinol (Lausanne). 2022 Mar 21;13:859347. doi: 10.3389/fendo.2022.859347 (PMC8977523; doi:10.3389/fendo.2022.859347)
Supplement: Supplementary file 1 [file DataSheet_1.docx]

Supplementary Material

# Supplementary Data

**1.1 Repeatability of the CLEIA**

We measured three types of urine samples, from low to high concentrations, 20 times in a row, and calculated the CVs based on the mean and standard deviation values. The CVs were 1.3–1.6% (Tables S1).

**1.2 Linearity of the CLEIA**

Four types of urine samples, from low to high concentrations, were diluted 10 times with diluent solution. Each dilution was tested in duplicate. Linearity was established via the double-measuring method in the range of 0.0 to 105354.0 pg/mL (Table S2, Fig. S1).

**1.3 Interference testing of the CLEIA**

Interference testing was performed by adding NaCl, Glucose, Alubmin, Creatinine, Ascorbic acid, Hemoglobin and Bilirubin C to pooled urine samples with low and high concentrations.

NaCl and Glucose were not affected at a concentration of 2.0 g/dL, Albumin was not affected at 1.0 g/dL, Creatinine was not affected at 400 mg/dL, Ascorbic acid was not affected 50 mg/dL,Hemoglobin was not affected at 500 mg/dL, and Bilirubin C was not affected at 19.7 mg/dL (Table S3).

Interference was calculated as follows:

% Interference = [Sample value (Interferent spiked) / Control Sample value (Blank)] x 100

**1.4 Effects of preservatives on CLEIA**

Testing the effects of preservatives on CLEIA was performed by adding Toluene, Xylene, Sodium azide to pooled urine samples with low and high concentrations. Toluene and Xylene were not affected at a concentration of 3.0%, Sodium azide was not affected at 1.0% (Table S4).

# Supplementary Figures and Tables

Table S1. Repeatability of the aldosterone measurements conducted in urine samples using CLEIA. Data are presented in pg/mL.

|  | Urine 1 | Urine 2 | Urine 3 |
| --- | --- | --- | --- |
| N | 20 | 20 | 20 |
| Mean | 3246.3 | 20415.9 | 80506.2 |
| SD | 52.3 | 256.1 | 1268.7 |
| CV | 1.6% | 1.3% | 1.6% |

Table S2. Linearity of aldosterone measured in urine samples using CLEIA.

(A) Linearity of aldosterone measured in urine samples using CLEIA in a concentration range of up to 96,000 pg/mL

(B) Linearity of aldosterone measured in urine samples using CLEIA over a concentration range of up to 96,000 pg/mL

| Dilution Rate | | Urine 1 | Urine 2 | Urine 3 |
| --- | --- | --- | --- | --- |
| Aldosterone  (pg/mL) | 0/10 | 0.0 | 0.0 | 0.0 |
|  | 1/10 | 294.0 | 1879.5 | 7719.0 |
|  | 2/10 | 618.0 | 3712.5 | 15529.5 |
|  | 3/10 | 912.0 | 5829.0 | 23086.5 |
|  | 4/10 | 1189.5 | 7654.5 | 31435.5 |
|  | 5/10 | 1543.5 | 9430.5 | 38559.0 |
|  | 6/10 | 1813.5 | 11169.0 | 46255.5 |
|  | 7/10 | 2191.5 | 13429.5 | 54712.5 |
|  | 8/10 | 2461.5 | 15060.0 | 63169.5 |
|  | 9/10 | 2844.0 | 17419.5 | 70518.0 |
|  | 10/10 | 3126.0 | 19072.5 | 77638.5 |
| Recovery  rate  　for 10/10 | 1/10 | 94.0% | 98.5% | 99.4% |
|  | 2/10 | 98.8% | 97.3% | 100.0% |
|  | 3/10 | 97.2% | 101.9% | 99.1% |
|  | 4/10 | 95.1% | 100.3% | 101.2% |
|  | 5/10 | 98.8% | 98.9% | 99.3% |
|  | 6/10 | 96.7% | 97.6% | 99.3% |
|  | 7/10 | 100.2% | 100.6% | 100.7% |
|  | 8/10 | 98.4% | 98.7% | 101.7% |
|  | 9/10 | 101.1% | 101.5% | 100.9% |
|  | 10/10 | 100.0% | 100.0% | 100.0% |

(A)

| Dilution Rate | | Urine 4 |
| --- | --- | --- |
| Aldosterone  (pg/mL) | 0/10 | 0.0 |
|  | 1/10 | 10714.5 |
|  | 2/10 | 20970.0 |
|  | 3/10 | 31561.5 |
|  | 4/10 | 42727.5 |
|  | 5/10 | 53602.5 |
|  | 6/10 | 62781.0 |
|  | 7/10 | 73086.0 |
|  | 8/10 | 84082.5 |
|  | 9/10 | 94947.0 |
|  | 10/10 | 105354.0 |
| Recovery  rate  　for 9/10 | 1/10 | 101.6% |
|  | 2/10 | 99.4% |
|  | 3/10 | 99.7% |
|  | 4/10 | 101.3% |
|  | 5/10 | 101.6% |
|  | 6/10 | 99.2% |
|  | 7/10 | 99.0% |
|  | 8/10 | 99.6% |
|  | 9/10 | 100.0% |
|  | 10/10 | 99.9% |

(B)

(A)

(B)

Fig S1. Linearity of aldosterone measured in urine samples using CLEIA.

(A) Linearity of aldosterone measured in urine samples using CLEIA in a concentration range of up to 96,000 pg/mL

(B) Linearity of aldosterone measured in urine samples using CLEIA over a concentration range of up to 96,000 pg/mL

Table S3. Interference in the aldosterone concentration data obtained using CLEIA.

| Interfering Substance | | Urine1 | | Urine2 | |
| --- | --- | --- | --- | --- | --- |
|  |  | Aldosterone Concentrations (pg/mL) | Interference  (%) | Aldosterone Concentrations (pg/mL) | Interference  (%) |
| NaCl (g/dL) | 0 | 2935.5 | 100.0% | 18358.5 | 100.0% |
|  | 0.4 | 2977.5 | 101.4% | 17847.0 | 97.2% |
|  | 0.8 | 3012.0 | 102.6% | 17976.0 | 97.9% |
|  | 1.2 | 2886.0 | 98.3% | 17461.5 | 95.1% |
|  | 1.6 | 2887.5 | 98.4% | 17470.5 | 95.2% |
|  | 2.0 | 2877.0 | 98.0% | 17608.5 | 95.9% |
| Glucose (g/dL) | 0 | 2943.0 | 100.0% | 18420.0 | 100.0% |
|  | 0.4 | 3010.5 | 102.3% | 18037.5 | 97.9% |
|  | 0.8 | 2818.5 | 95.8% | 17391.0 | 94.4% |
|  | 1.2 | 2857.5 | 97.1% | 17952.0 | 97.5% |
|  | 1.6 | 2868.0 | 97.5% | 18078.0 | 98.1% |
|  | 2.0 | 2878.5 | 97.8% | 18012.0 | 97.8% |
| Albumin (g/dL) | 0 | 2965.5 | 100.0% | 17877.0 | 100.0% |
|  | 0.2 | 2877.0 | 97.0% | 17458.5 | 97.7% |
|  | 0.4 | 2995.5 | 101.0% | 17832.0 | 99.7% |
|  | 0.6 | 2817.0 | 95.0% | 17947.5 | 100.4% |
|  | 0.8 | 2917.5 | 98.4% | 17814.0 | 99.6% |
|  | 1.0 | 2952.0 | 99.5% | 17916.0 | 100.2% |
| Creatinine (mg/dL) | 0 | 2991.0 | 100.0% | 18024.0 | 100.0% |
|  | 80 | 2871.0 | 96.0% | 18219.0 | 101.1% |
|  | 160 | 2989.5 | 99.9% | 18018.0 | 100.0% |
|  | 240 | 2917.5 | 97.5% | 18030.0 | 100.0% |
|  | 320 | 2974.5 | 99.4% | 18103.5 | 100.4% |
|  | 400 | 2892.0 | 96.7% | 17671.5 | 98.0% |
| Ascorbic acid (mg/dL) | 0 | 2968.5 | 100.0% | 17628.0 | 100.0% |
|  | 10 | 2938.5 | 99.0% | 17578.5 | 99.7% |
|  | 20 | 2857.5 | 96.3% | 17943.0 | 101.8% |
|  | 30 | 2814.0 | 94.8% | 17848.5 | 101.3% |
|  | 40 | 2863.5 | 96.5% | 17587.5 | 99.8% |
|  | 50 | 2859.0 | 96.3% | 17683.5 | 100.3% |
| Hemoglobin (mg/dL) | 0 | 2974.5 | 100.0% | 18015.0 | 100.0% |
|  | 100 | 2851.5 | 95.9% | 17374.5 | 96.4% |
|  | 200 | 2800.5 | 94.2% | 17551.5 | 97.4% |
|  | 300 | 2850.0 | 95.8% | 17314.5 | 96.1% |
|  | 400 | 2836.5 | 95.4% | 17265.0 | 95.8% |
|  | 500 | 2910.0 | 97.8% | 17967.0 | 99.7% |
| Bilirubin C (mg/dL) | 0 | 2997.0 | 100.0% | 18163.5 | 100.0% |
|  | 3.9 | 2962.5 | 98.8% | 17988.0 | 99.0% |
|  | 7.9 | 2917.5 | 97.3% | 17466.0 | 96.2% |
|  | 11.8 | 2911.5 | 97.1% | 17749.5 | 97.7% |
|  | 15.8 | 2815.5 | 93.9% | 18273.0 | 100.6% |
|  | 19.7 | 2835.0 | 94.6% | 17653.5 | 97.2% |

Table S4. Effect of preservatives in the aldosterone concentration data obtained using CLEIA.

| Preservatives | | Urine1 | | Urine2 | |
| --- | --- | --- | --- | --- | --- |
|  |  | Aldosterone Concentrations (pg/mL) | Interference  (%) | Aldosterone Concentrations (pg/mL) | Interference  (%) |
| Toluene (%) | 0 | 2964.0 | 100.0% | 17352.0 | 100.0% |
|  | 0.6 | 2853.0 | 96.3% | 17686.5 | 101.9% |
|  | 1.2 | 2932.5 | 98.9% | 17836.5 | 102.8% |
|  | 1.8 | 2829.0 | 95.4% | 17769.0 | 102.4% |
|  | 2.4 | 2847.0 | 96.1% | 17670.0 | 101.8% |
|  | 3.0 | 2817.0 | 95.0% | 17458.5 | 100.6% |
| Xylene (%) | 0 | 2953.5 | 100.0% | 18106.5 | 100.0% |
|  | 0.6 | 2923.5 | 99.0% | 17986.5 | 99.3% |
|  | 1.2 | 2896.5 | 98.1% | 17796.0 | 98.3% |
|  | 1.8 | 2895.0 | 98.0% | 17769.0 | 98.1% |
|  | 2.4 | 2889.0 | 97.8% | 17731.5 | 97.9% |
|  | 3.0 | 2871.0 | 97.2% | 17821.5 | 98.4% |
| Sodium azide (%) | 0 | 2997.0 | 100.0% | 18093.0 | 100.0% |
|  | 0.2 | 2886.0 | 96.3% | 18163.5 | 100.4% |
|  | 0.4 | 2877.0 | 96.0% | 18321.0 | 101.3% |
|  | 0.6 | 2841.0 | 94.8% | 18477.0 | 102.1% |
|  | 0.8 | 2919.0 | 97.4% | 18163.5 | 100.4% |
|  | 1.0 | 2988.0 | 99.7% | 18814.5 | 104.0% |
